# Nutrient use efficiency (NUE) of wheat (*Triticum aestivum* L.) as affected by NPK fertilization

**DOI:** 10.1371/journal.pone.0262771

**Published:** 2022-01-27

**Authors:** Nabin Rawal, Keshab Raj Pande, Renuka Shrestha, Shree Prasad Vista

**Affiliations:** 1 Department of Soil Science and Agri-Engineering, Agriculture and Forestry University, Rampur, Chitwan, Bagmati, Nepal; 2 National Soil Science Research Centre, Nepal Agricultural Research Council, Khumaltar, Lalitpur, Bagmati, Nepal; 3 National Agronomy Research Centre, Nepal Agricultural Research Council, Lalitpur, Bagmati, Nepal; Mendel University of Agriculture and Forestry: Mendelova univerzita v Brne, CZECH REPUBLIC

## Abstract

Nutrient use efficiency is crucial for increasing crop yield and quality while reducing fertilizer inputs and minimizing environmental damage. The experiments were carried out in silty clay loam soil of Lalitpur, Nepal, to examine how different amounts of nitrogen (N), phosphorus (P), and potassium (K) influenced crop performance and nutrient efficiency indices in wheat during 2019/20 and 2020/21. The field experiment comprised three factorial randomized complete block designs that were replicated three times. N levels (100, 125, 150 N kg ha^-1^), P levels (25, 50, 75 P_2_O_5_ kg ha^-1^), and K levels (25, 50, 75 K_2_O kg ha^-1^) were three factors evaluated, with a total of 27 treatment combinations. Grain yields were significantly increased by N and K levels and were optimum @ 125 kg N ha^-1^ and @ 50 kg K_2_O ha^-1^ with grain yields of 6.33 t ha^-1^ and 6.30 t ha^-1^, respectively. Nutrient levels influenced statistically partial factor productivity, internal efficiency, partial nutrient budget, recovery efficiency, agronomic efficiency, and physiological efficiency of NPK for wheat. Nutrient efficiency was found to be higher at lower doses of their respective nutrients. Higher P and K fertilizer rates enhanced wheat N efficiencies, and the case was relevant for P and K efficiencies as well. Wheat was more responsive to N and K fertilizer, and a lower rate of P application reduced N and K fertilizer efficiency. This study recommends to use N @ 125 kg ha^-1^, P_2_O_5_ @ 25 kg ha^-1^ and K_2_O @ 50 kg ha^-1^ as an optimum rate for efficient nutrient management in wheat in mid-hills of Nepal.

## Introduction

For more than 35% of the world’s population, wheat (*Triticum aestivum* L.) is the primary source of nutrition [1], providing more than 45% of calories and more than 40% of protein to the world’s population [2]. In Nepal, wheat ranks as third major crops which is being cultivated on 707,505 ha, producing 2,185,289 metric tons with a low productivity of 3.09 t ha^-1^ [3] as compared to other developed countries such as China (5.63 t ha^-1^) [FAOSTAT; www.fao.org]. At the same time, the wheat demand would rise in Nepal with increasing population, indicating necessity to increase the wheat yield through various management approaches [4]. Nutrition management is one of approach to improve the crop yield [5]. Wheat depletes soil nutrients, so if it isn’t adequately fertilized, soil fertility starts to decline [6]. Therefore, fertilizer applications are essential to maintain a positive nutrient balance by replacing nutrients that are taken and lost during cropping [7]. However, increasing nutrient use efficiency (NUE) is critical in order to achieve expected production while using as little fertilizer as possible. The use of the proper fertilizer in the right amount is one of the most important management strategies for increasing fertilizer efficiency [8] and maximize crop productivity [9]. The application of synthetic fertilizers in wheat field increases available nitrogen, phosphorus, and potassium in soil [10]. Optimum dose of fertilizer improves wheat yield [11] and fertilizer use efficiency, and reducing pollution [5, 12]. Moreover, right combination of primary nutrients is also important to enhance wheat yield and NUE [13]. In cereal crops, the global N use efficiency was found to be 33% [14]. NUE declines with increased N dose [15–17] while crop production increases [15]. [16] reported a lower nitrogen use efficiency (27.1%) from a nitrogen rate of 120 kg N ha^-1^ compared to a nitrogen rate of 30 kg N ha^-1^ with 39.27% nitrogen use efficiency. One of the reasons for lower nitrogen efficiency is N losses, limits only 50% of applied nitrogen fertilizer available to cereal crops [18]. The average Recovery efficiency of nitrogen (RE_N_) for wheat in worldwide research trials is 57% [19], but wheat recovery efficiency values for N ranged from 50 to 80 kg kg^-1^ in a well-managed system with minimal N application and poor soil N supply [20]. The global P use efficiency was reported to be 16% in cereal crops [21]. Most agricultural crops recovered 20–30% of applied P during their growth under suitable growing environments [22].

Many studies have demonstrated that potassium fertilizer efficacy is dependent on optimal N and P levels; efficacy is lower when potassium fertilizer is applied alone or with P only, but higher when potassium fertilizer is applied with N [23]. Potassium deficiency has been observed in extensively cultivated soils in Nepal as a result of inadequate replenishment [24]. As a consequence, the application of potassium fertilizer has been responding well [25]. In high-hills conditions of Nepal, 45 to 60 kg ha^-1^ produced significantly better grain yields than the recommended K dose [26]. At the lowest K rate (30 kg K_2_O ha^-1^), the maximum apparent K recovery and agronomic usage efficiency were attained [27]. Similarly, when K levels rise, wheat apparent recovery and agronomic use efficiency decline [28]. Under ideal conditions, an achievable range of 40–60% potassium recovery efficiency has been recorded in crops cultivated in soils with low potassium content [22].

Aside from the individual effects of nutrients, the interaction of nutrients is also crucial for yield and nutrient efficiency. Nitrogen aids in the efficient utilization of potassium, phosphorus, and other nutrients by plants [29]. N and P use efficiency, as well as productivity and quality of agriculture produce, could all benefit from increased K fertilization [30]. For wheat cultivated in typical South Asian soils low in organic C, the average recovery effectiveness of N, P, and K applied 120 kg N ha^-1^, 26 kg P ha^-1^, and 50 kg K ha^-1^ was 58%, 27%, and 51%, respectively [31]. AE_P_ in wheat ranged from 22 to 63 kg grain kg^-1^ P when averaged across N rates, whereas RE_P_ varied from 22 to 40% [32].

Fertilizer use in Nepal is not only inadequate, but it is also applied in an unbalanced manner [11]. Farmers in Nepal usually apply N and P-containing fertilizer as a blanket recommendation, while K-containing fertilizer is not commonly applied. This is one of the factors contributing to low fertilizer use efficiency in Nepal. Moreover, plant’s potential for extracting nutrients from the soil and its efficient use within the plant system has been poorly understood in case of wheat in Nepal. Therefore, the best way of using nutrients, such as the optimum dose, should be evaluated and investigated in order to minimize losses and achieve improve fertilizer practices [33]. Improved nutrient use efficiency will not only help to lower the cost of crop production by reducing fertilizer use, but also help to reduce fertilizer contamination [34]. Despite the fact that using less fertilizer increases nutrient use efficiency, farmers are concerned about optimizing profit [35]. So, it’s essential to find a balance between nutrient efficiency and crop productivity. To address these gaps, this study aims to find best combination of nitrogen, phosphorous and potassium as well as nutrient use efficiency in wheat for improve nutrient management strategy in Nepal.

## Materials and methods

### Characteristics of experimental site

**Fig 1:**
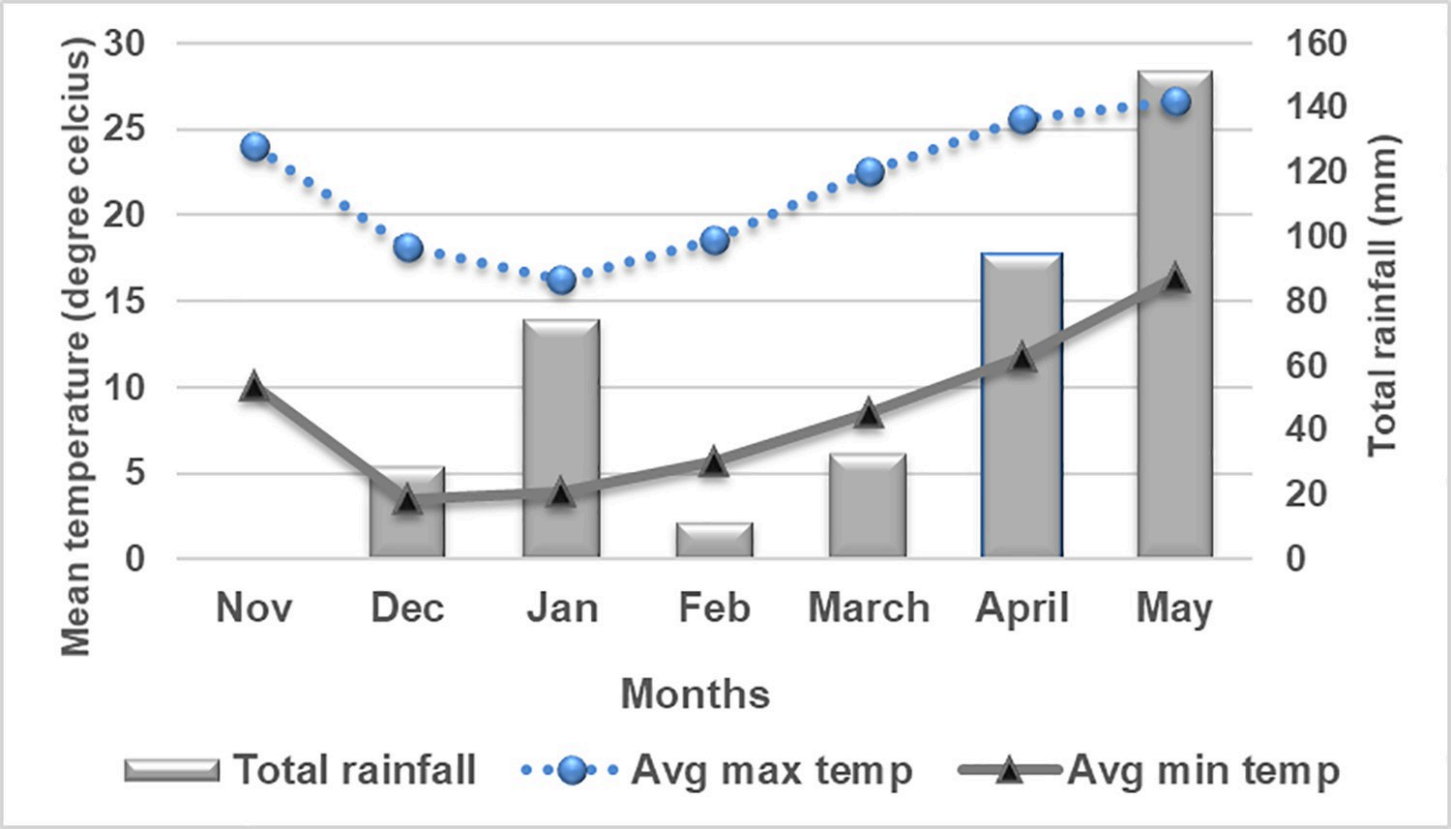
Meteorological data of National Agronomy Research Centre, Khumaltar, Lalitpur, Nepal during Nov-May, 2019/20.

**Fig 2:**
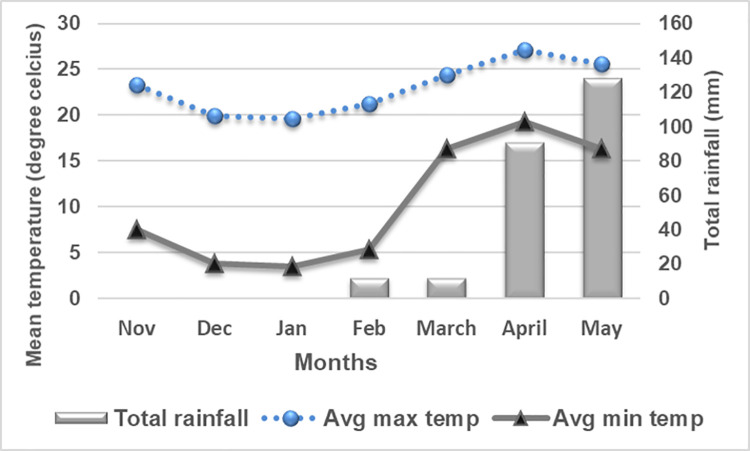
Meteorological data of National Agronomy Research Centre, Khumaltar, Lalitpur, Nepal during Nov-May, 2020/21.

Field experiments were conducted at National Agronomy Research Centre during winters of 2019/20 and 2020/21 and laboratory work were carried out at National Soil Science Research Centre, Khumaltar, Lalitpur, Nepal. The location falls in the mid hill valley condition of Nepal (27^0^39’ N, 85^0^19’ E, 1285 masl). The climate is sub-tropical with hot and dry summers, cold winters and monsoon rains generally starts in July and continue till October. The average annual rainfall is 1347.6 mm, 74% of rainfall receiving between the months of June and October. Meteorological data on rainfall, and temperature received from the National Agronomy Research Centre, Khumaltar, Lalitpur, Nepal for crop seasons (July 2013 to June 2015) is presented below (Figs 1 and 2). The initial soil fertility status, particularly a pH of 5.98 (acidic), low organic matter (2.01%), medium total nitrogen (0.14%), high available P_2_O_5_ (478.6 kg ha^-1^), medium available K_2_O (160.5 kg ha^-1^), with a silty clay loam soil texture and the average bulk density of 1.39 gm cm^-3^ was estimated in laboratory at the beginning of the experiment and graded using soil value chart [36].

### Experimental treatments and setup

Three factorial randomized complete block design (RCBD) with 27 treatments and three replications were used in the study with plot size of 10.5 m^2^ (4.2 m x 2.5 m). The experiment comprised of three factors, three levels of nitrogen (100, 125, and 150 kg ha^-1^), phosphorus (25, 50, and 75 kg ha^-1^), and potassium (25, 50, and 75 kg ha^-1^), with total 81 plots. Three replications of three nutritional omission treatments (-N, -P, and -K) were also included in the treatments to measure NUE especially recovery efficiency of wheat.

### Crop management

In the experiment, the pipeline wheat genotype WK-2286 was used which was provided by National Plant Breeding and Genetics Research Centre, Khumaltar, Lalitpur, Nepal. Wheat seed was sown in well-prepared plots in a 25 cm row with continuous sowing on the 14th of November 2019 and the 10^th^ of November 2020. Urea (46% N), single super phosphate (16% P_2_O_5_), and muriate of potash (60% K_2_O) were used to supply nitrogen, phosphorus, and potassium. Phosphorus and potassium fertilizers were applied at full amounts as a basal application during final land preparation, while nitrogen was applied in three phases: 1/3 as a basal application, 1/3 at maximum tillering, and 1/3 at the panicle initiation stages of wheat. The experiment was managed using all of the recommended wheat production cultural practices consistently as required. The crop was manually harvested on 12^th^ May, 2020, and 17^th^ May, 2021.

### Plant sampling and analysis

When the crop reached maturity, grain yield was measured on a subplot basis using a net plot area of 5.25 m^2^, adjusted for moisture content at 12% and converted to tons per hectare. Wheat plants were randomly cut at ground level as destructive samples to quantify dry matter accumulation in each plot. The sample was washed with distilled water to remove attached soil and dust. The harvested grain and straw samples were oven dried at 70°C until consistent weights, then grinded to a fine powder that passed through a 0.2mm sieve and a prepared 10 g sample was packed in polythene bags. The samples were analyzed for N, P and K as per the standard procedures. For N determination, 0.2 g of grain and straw samples were digested with concentrated sulfuric acid and a digestion mixture (K_2_SO_4_: CuSO_4_.5H_2_O: Selenium in a 100: 10: 1 proportion) until a green residue was formed. Kjeldahl distillation method was used to distill the digested solution [37]. The nitrogen was measured using a titration against standard sulfuric acid after the ammonia was captured in 4% boric acid [38]. For P and K determination, 0.5 g of grain and straw samples were digested in a di-acid of concentrated nitric acid (HNO_3_) and hydrogen per oxide (H_2_O_2_). From an extract obtained from acid digestion, P and K were measured using the Vanadate-Molybdate-phosphoric yellow color method and the flame photometry method, respectively [38]. By multiplying the nutrient concentrations (%) by the respective straw and grain production (kg ha^-1^), nutrient uptake by the straw and grain was estimated. The total nutrient uptake by the whole plant was calculated by adding grain and straw nutrient uptake.

### Calculation of nutrient efficiency indices

To evaluate nutrient use efficiencies (NUE) of applied fertilizers, partial factor productivity (PFP), internal efficiency (IE), partial nutrient budget (PNB), recovery efficiency (RE), agronomic efficiency (AE) and physiological efficiency (PE) are typically adopted [22, 39–41].

The Partial Factor Productivity is calculated as crop yield (kg) per unit of applied nutrient (kg).

${\mathrm{PFP}}_{X}=Y_{A}/F_{A}$ [5, 40–42]

Internal efficiency is the quantity of grain yield produced per kilogram of total nutrient accumulation in aboveground plant dry matter. A high IUE indicates nutrient deficiency, whereas a low IUE indicates inefficient internal nutrient conversion due to various stresses such as deficiencies of other nutrients, drought stress, heat stress, mineral toxicities, pests, etc. [40]

${\mathrm{IE}}_{X}=Y_{A}/U_{A}$ [22, 40, 41]

The Partial Nutrient Budget is a tool for determining a cropping system’s long-term viability in terms of nutrient uptake by the harvested component per unit of applied nutrient [39].

${\mathrm{PNB}}_{X}=U_{A}/F_{A}$ [40, 41]

Increased grain yield (kg) per unit of applied fertilizer (kg) is how agronomic efficiency is measured.

${\mathrm{AE}}_{X}={(Y}_{A}{-Y}_{O})/F_{A}$ [22, 40–42]

Recovery efficiency refers to the increase in crop uptake of a nutrient in the aboveground parts of the plant as a result of its application. Scientists evaluating the nutrient response of the crop frequently prefer NUE expression.

${\mathrm{RE}}_{X}={(U}_{A}{-U}_{O})\times 100/F_{A}$ [22, 40–42]

The ratio of kg grain yield to kg nutrient uptake in above-ground dry matter production is known as physiological efficiency.

${\mathrm{PE}}_{X}={(Y}_{A}{-Y}_{O})/{(U}_{A}{-U}_{O})$ [22, 42]

Where, X = N, P, and K; Y_A_ = grain yield with nutrient applied; Y_O_ = grain yield with no nutrient applied; U_A_ = total nutrient uptake with nutrient applied; U_O_ = total nutrient uptake with no nutrient applied; F_A_ = with fertilizer; F_0_ = without fertilizer. PFP, IE, PNB, AE, PE are expressed in kg kg^-1^ whereas RE is expressed in percentage. All fertilizers used, crop yield, and total nutrient uptake are all measured in kilograms.

### Statistical analysis

Both years (2019/20 and 2020/21) grain yield and NUE indices were averaged to calculate pooled mean of grain yield and NUE. Data were subjected to analysis of variance (ANOVA) to determine a significant difference of treatment impact at a 5% level of significance and Duncan’s Multiple Range Test was used for mean separation [43]. GenStat (Version 18.0) was used for running statistically analysis. Microsoft Excel 10.0 and Sigma plot software (version 12.0) were used to generate the graphs. Bar diagram was drawn using mean value and confidence interval (95%) of the treatments.

## Results

### Wheat yield

**Fig 3:**
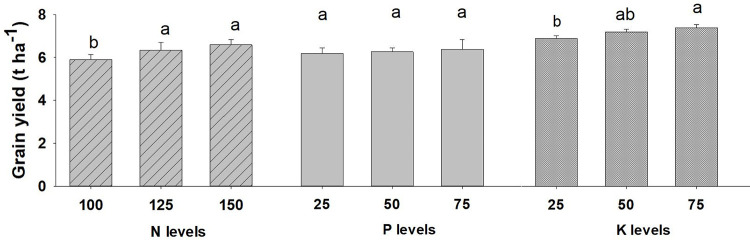
Wheat grain yield as affected by NPK levels (2019/20-2020/21). Different small alphabetical letters indicate significant differences at p<0.05 (otherwise statistically at par). Bars indicate mean value of treatments with 95% confidence interval.

The interaction effect of N, P and K were found to be non-significant on the grain yield of wheat, as given in Table 1. The N and P levels had significant effect on the pooled mean on grain yield of wheat (Fig 3). Wheat grain yield was significantly higher with the application of N @ 150 kg ha^-1^ (6.59 t ha^-1^) which was at par with N @ 125 kg ha^-1^ (6.33 t ha^-1^). The lowest grain yield was recorded with N @ 100 kg ha^-1^. Similarly, K_2_O @ 75 kg ha^-1^ produced statistically higher grain yields (6.48 t ha^-1^) followed by K_2_O @ 50 kg ha^-1^ (6.30 t ha^-1^). The lowest grain yield of 6.04 t ha^-1^ was produced by K_2_O @ 25 kg ha^-1^. However, there was no significant response of phosphorus level on grain yield beyond 25 kg P_2_O_5_ kg ha^-1^ though the highest grain yield of 6.38 t ha^-1^ were measured with application of P_2_O_5_ @ 75 kg ha^-1^.

**Table 1:** Summary of analysis of variance (ANOVA) of F test probability (P>F) (pooled data of two years).

Parameters	Nutrient	N	P	K	N x P	N x K	P x K	N x P x K	Year
**Grain yield**		***	NS	**	NS	NS	NS	NS	
**PFP**	N	***	NS	**	NS	NS	NS	NS	**
	P	***	***	**	NS	NS	NS	NS	**
	K	***	NS	***	NS	*	NS	NS	**
**IE**	N	***	NS	*	NS	NS	NS	*	**
	P	***	**	NS	NS	NS	NS	NS	**
	K	***	NS	**	NS	NS	NS	NS	**
**PNB**	N	***	*	***	*	NS	NS	NS	**
	P	***	***	*	**	NS	NS	NS	**
	K	***	NS	***	NS	*	NS	NS	NS
**RE**	N	NS	*	**	NS	NS	NS	NS	***
	P	***	**	*	*	NS	NS	NS	NS
	K	***	NS	**	NS	NS	NS	NS	NS
**AE**	N	***	NS	**	NS	NS	NS	NS	**
	P	***	***	**	*	NS	NS	NS	*
	K	***	NS	**	NS	*	NS	NS	**
**PE**	N	***	NS	*	NS	NS	NS	NS	**
	P	*	*	*	NS	NS	NS	NS	**
	K	*	NS	**	NS	NS	NS	NS	NS

NS = non-significant (p>-0.05)

*** = significant at 0.1%

* = significant at 1% and * = significant at 5%.

### Nutrient use efficiencies indices

#### Partial factor productivity

**Fig 4:**
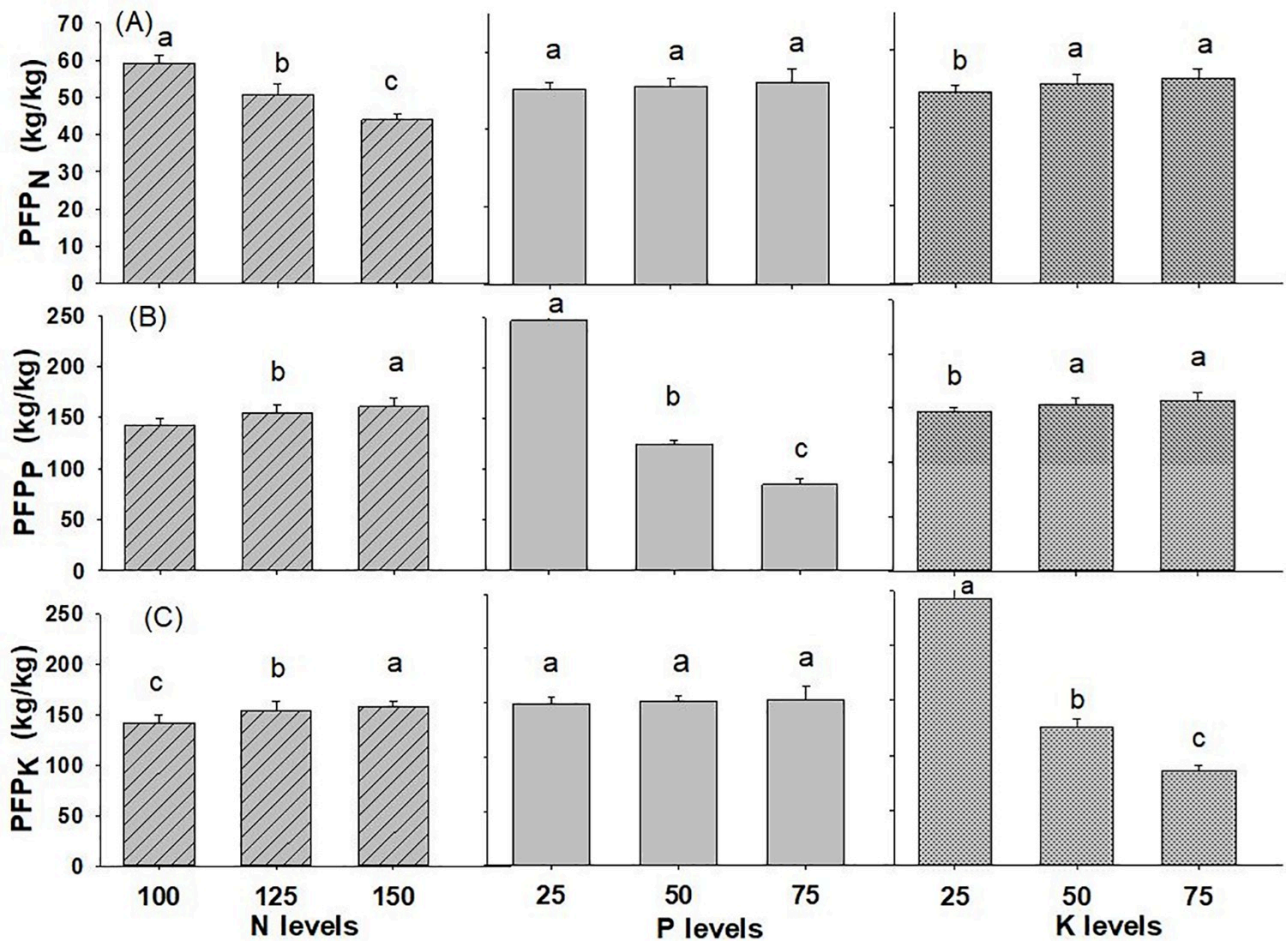
Partial factor productivity of wheat (A) nitrogen (B) phosphorus (C) potassium as influenced by different levels of NPK fertilizer (2019/20-2020/21). Different small alphabetical letters indicate significant differences at p<0.05 (otherwise statistically at par). Bars indicate mean value of treatments with 95% confidence interval.

The interaction of N x K on the partial factor productivity of potassium in wheat crop was found to be significant and all the other interaction effects of N, P and K were found to be on par with each other, as reported in Table 1 and S1 Table. The PFP_N_ decline significantly with increase in rate of N and measured 59.0 kg kg^-1^ with N @ 100 kg ha^-1^ to 43.9 kg kg^-1^ with N @ 150 kg ha^-1^ applied to the soil (Fig 4). However, PFP_N_ increased non-significantly with higher P_2_O_5_ levels and significantly higher with K_2_O application rate from 25 to 50 kg ha^-1^. PFP_P_ increased significantly with higher dose of N (from 100 to 150 kg ha^-1^), and K_2_O (from 25 to 50 kg ha^-1^), however, the trend is opposite with higher levels of P_2_O_5_ applied to soil. The highest PFP_P_ was 160.6 kg kg^-1^, 247.3 kg kg^-1^ and 157.4 kg kg^-1^ at N @ 150 kg ha^-1^, P_2_O_5_ @ 25 kg ha^-1^ and K_2_O @ 75 kg ha^-1^. PFP_K_ also increased significantly with higher levels of N and opposite for K levels. The PFP_K_ was found non-significant under levels of P_2_O_5._ The highest PFP_K_ was 158.6 kg kg^-1^, 153.1 kg kg^-1^, 241.6 kg kg^-1^ at N @ 150 kg ha^-1^, P_2_O_5_ @ 75 kg ha^-1^ and K_2_O @ 25 kg ha^-1^, respectively.

#### Internal efficiency

**Fig 5:**
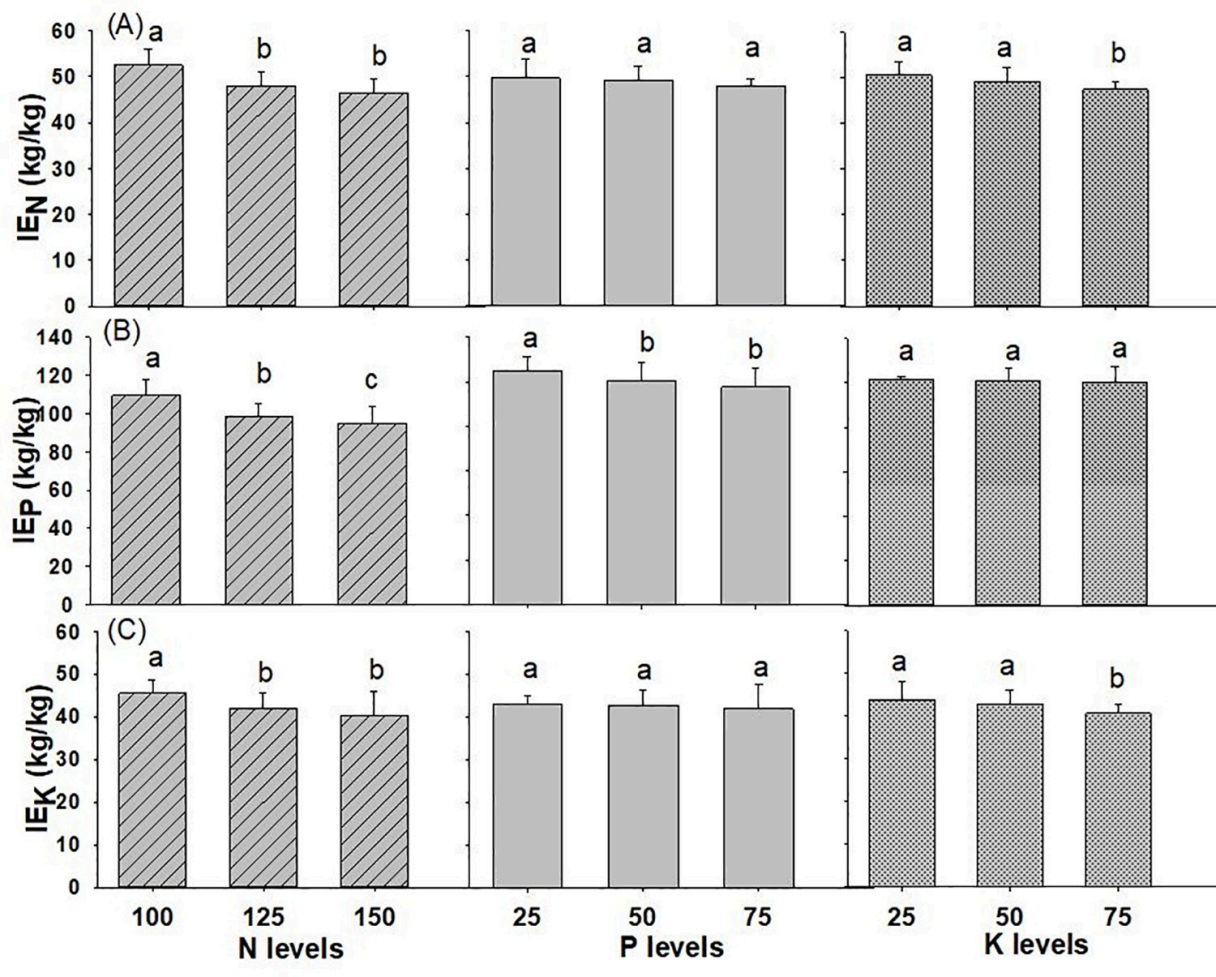
Internal Efficiency of wheat (A) nitrogen (B) phosphorus (C) potassium as influenced by different levels of NPK fertilizer (2019/20-2020/21). Different small alphabetical letters indicate significant differences at p<0.05 (otherwise statistically at par). Bars indicate mean value of treatments with 95% confidence interval.

The interaction effects of N, P and K on the internal efficiency of nutrients in wheat crop was found to be on par with each other, as shown in Table 1 and S1 Table. However, the IE_N_ declined significantly with increase in rate of N and measured 52.6 kg kg^-1^ with N @ 100 kg ha^-1^ to 46.5 kg kg^-1^ with N @ 150 kg ha^-1^ applied to the soil (Fig 5). Similarly, IE_N_ decreased non-significantly with higher P levels and statistically lower with K_2_O application rate from 25 to 75 kg ha^-1^. IE_P_ decreased remarkably with higher dose of N (from 100 to 150 kg ha^-1^), and P_2_O_5_ (from 25 to 75 kg ha^-1^), however, the effect was non-significant with application of K levels. The highest IE_P_ was 109.5 kg kg^-1^, 104.8 kg kg^-1^ and 101.5 kg kg^-1^ with the application of N @ 100 kg ha^-1^, P_2_O_5_ @ 25 kg ha^-1^ and K_2_O @ 25 kg ha^-1^. PFP_K_ also decreased significantly with higher levels of N and for K levels but it was found non-significant under levels of P_2_O_5._ The highest IE_K_ was 45.4 kg kg^-1^, 43.02 kg kg^-1^, 43.9 kg kg^-1^ with the use of N @ 100 kg ha^-1^, P_2_O_5_ @ 25 kg ha^-1^ and K_2_O @ 25 kg ha^-1^, respectively.

#### Partial nutrient budget

**Fig 6:**
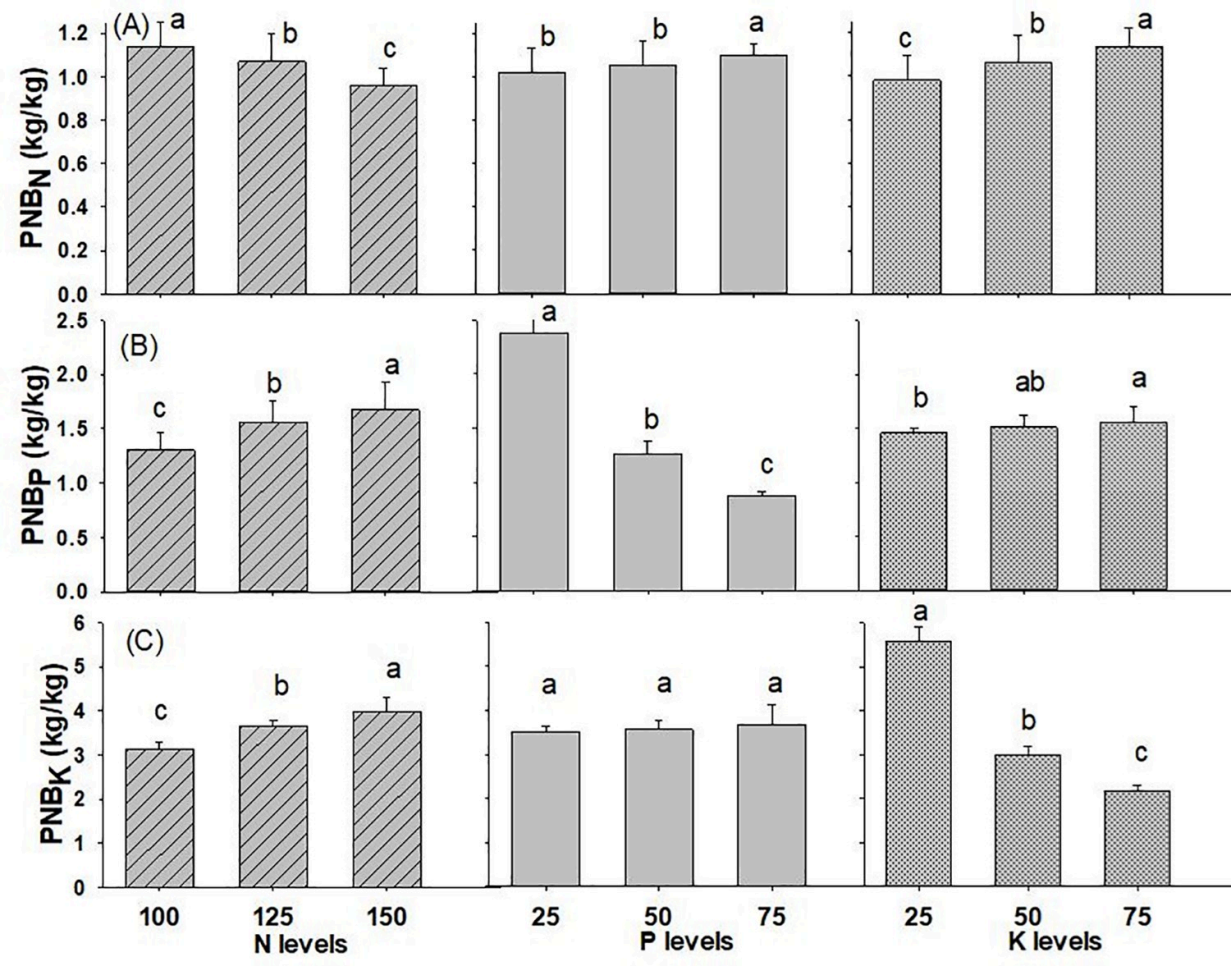
Partial Nutrient Budget of wheat (A) nitrogen (B) phosphorus (C) potassium as influenced by different levels of NPK fertilizer (2019/20-2020/21). Different small alphabetical letters indicate significant differences at p<0.05 (otherwise statistically at par). Bars indicate mean value of treatments with 95% confidence interval.

The interaction of N x P on PNB_N_ and PNB_P_ and interaction of N x K on PNB_K_ was found significant in wheat crop while all the other interaction effects of N, P and K on partial nutrient budget of wheat were found to be on par with each other, as presented in Table 1 and S1 Table. (The effect of nitrogen levels on PNB_N_ in wheat was substantially higher with a value of 1.14 kg kg^-1^ when N @ 100 kg ha^-1^ was applied, and the lowest value of 0.96 kg kg^-1^ when N @ 150 kg ha^-1^ was applied (Fig 6). Likewise, using P_2_O_5_ @ 75 kg ha^-1^ and K_2_O @ 75 kg ha^-1^, yielded higher values of PNB_N_ (1.10 kg kg^-1^and 1.13 kg kg^-1^, respectively), while using P_2_O_5_ @ 25 kg ha^-1^ and K_2_O @ 25 kg ha^-1^ yielded the lowest values of 1.02 kg kg^-1^ and 0.98 kg kg^-1^, respectively. In the same way, the application of N @ 150 kg ha^-1^ and K_2_O @ 75 kg ha^-1^ resulted in considerably greater PNB_P_ (1.67 kg kg^-1^ and 1.56 kg kg^-1^), respectively), whereas the application of N @ 100 kg ha^-1^ and K_2_O @ 25 kg ha^-1^ resulted in significantly lower PNB_P_ of 1.30 kg kg^-1^ and 1.46 kg kg^-1^, respectively. The treatment of P_2_O_5_ @ 25 kg ha^-1^ observed a statistically higher PNB_P_ value (2.38 kg kg^-1^), whereas the application of P_2_O_5_ @ 75 kg ha^-1^ recorded the lowest value of 0.88 kg kg^-1^. Similarly, wheat crop applied with N @ 150 kg ha^-1^ and P_2_O_5_ @ 75 kg ha^-1^ had considerably higher PNB_K_ of 3.96 kg kg^-1^ and 3.68 kg kg^-1^, respectively, while wheat crop applied with N @ 100 kg ha^-1^ and P_2_O_5_ @ 25 kg ha^-1^, had the lowest values of PNB_K_ (3.13 kg kg^-1^ and 3.50 kg kg^-1^), respectively. When K_2_O @ 25 kg ha^-1^ was supplied, the effect on PNB_K_ in wheat was much larger (5.59 kg kg^-1^) and was lower (2.16 kg kg^-1^) when K_2_O @ 80 kg ha^-1^ was applied in the soil.

#### Recovery efficiency

**Fig 7:**
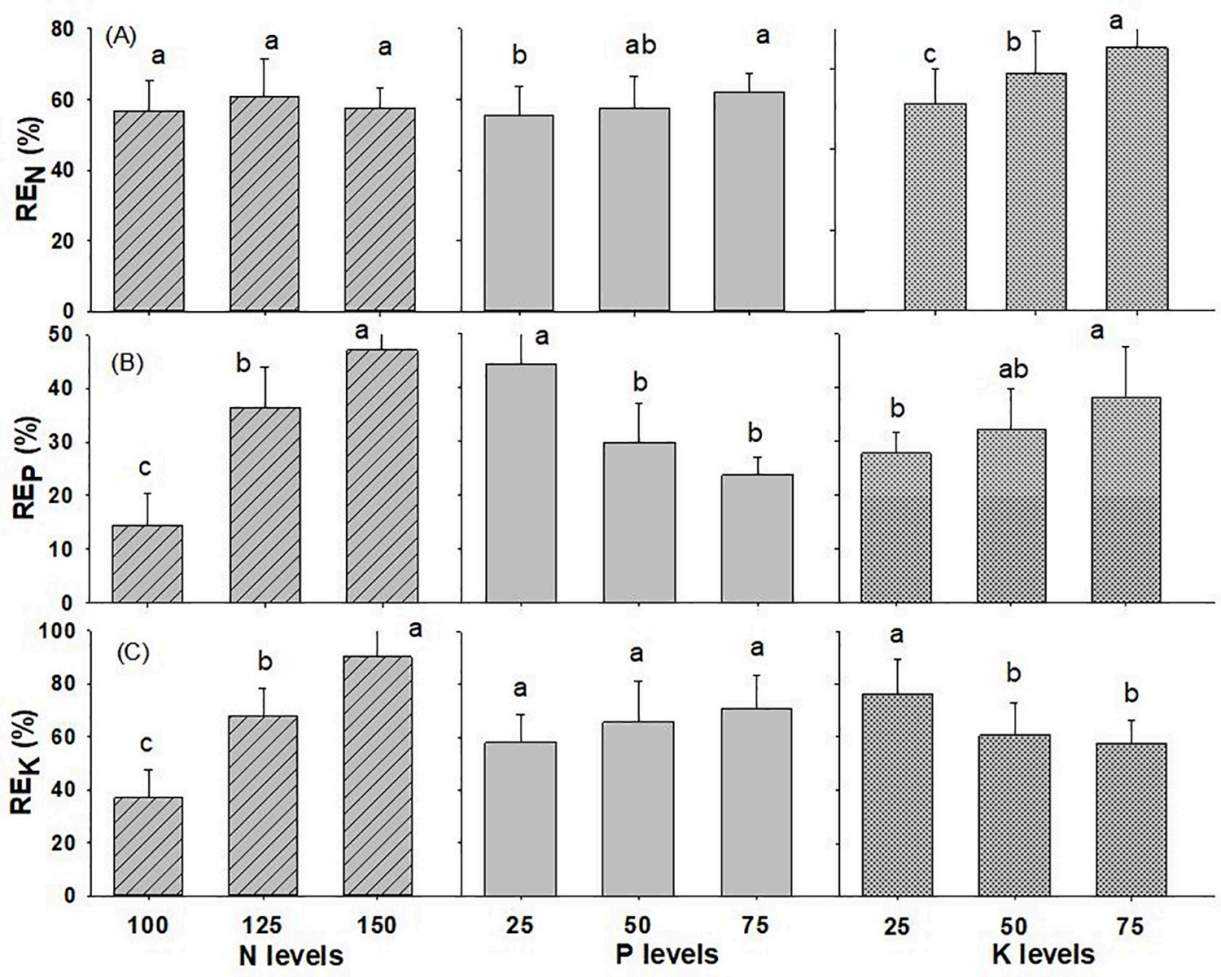
Recovery Efficiency of wheat (A) nitrogen (B) phosphorus (C) potassium as influenced by different levels of NPK fertilizer (2019/20-2020/21). Different small alphabetical letters indicate significant differences at p<0.05 (otherwise statistically at par). Bars indicate mean value of treatments with 95% confidence interval.

The interaction of N x P on the recovery efficiency of phosphorus was found to be significant and all the other interaction effects of N, P and K were found to be at par with each other in wheat crop, as shown in Table 1 and S2 Table. The application of N @ 125 kg ha^-1^, P_2_O_5_ @ 75 kg ha^-1^ and K_2_O @ 75 kg ha^-1^ produced the highest RE_N_ of 60.9%, 62.2%, and 65.2%, respectively), while N @ 100 kg ha^-1^, P_2_O_5_ @ 25 kg ha^-1^ and K_2_O @ 25 kg ha^-1^ gave the lowest RE_N_ (56.8%, 55.3%, and 51.3%, respectively) (Fig 7). In the same way, greater RE_P_ of 47.1% was obtained with the application of 150 kg N ha^-1^, 44.4% with the application of 25 kg P_2_O_5_ ha^-1^, and 38.1 with the application of K_2_O @ 75 kg ha^-1^, while lower RE_P_ was obtained with the application of N @ 100 kg ha^-1^, P_2_O_5_ @ 75 kg ha^-1^ and K_2_O @ 25 kg ha^-1^ (14.5%, 23.7% and 27.7%, respectively). Similarly, higher RE_K_ of 89.9%, 70.8%, and 76.0% were observed with the use of N @ 150 kg ha^-1^, P_2_O_5_ @ 75 kg ha^-1^ and K_2_O @ 25 kg ha^-1^, respectively, whereas the lowest values of RE_K_ (36.8%, 58.1%, and 57.7%) were obtained with the use of N @ 100 kg ha^-1^, P_2_O_5_ @ 25 kg ha^-1^ and K_2_O @ 75 kg ha^-1^, respectively.

#### Agronomic efficiency

**Fig 8:**
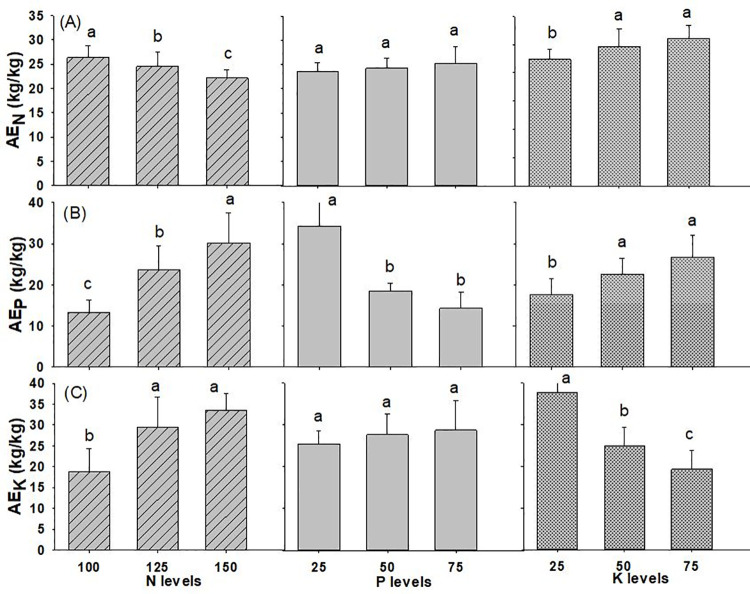
Agronomic Efficiency of wheat (A) nitrogen (B) phosphorus (C) potassium as influenced by different levels of NPK fertilizer (2019/20-2020/21). Different small alphabetical letters indicate significant differences at p<0.05 (otherwise statistically at par). Bars indicate mean value of treatments with 95% confidence interval.

The interactions of N x P on AE_P_ and N x K on AE_K_ were found significant in wheat crop while all the other interaction effects of N, P and K on agronomic efficiency of wheat were found to be on par with each other, as reported in Table 1 and S2 Table. The AE_N_ decline significantly with increase in rate of N and measured 26.4 kg kg^-1^ with N @ 100 kg ha^-1^ to 22.1 kg kg^-1^ with N @ 150 kg ha^-1^ applied to the soil (Fig 8). However, AE_N_ increased non-significantly with higher P levels and significantly higher with K_2_O application rate from 25 to 50 kg ha^-1^. AE_P_ increased significantly with higher dose of N (from 100 to 150 kg ha^-1^), and K_2_O (from 25 to 50 kg ha^-1^), however, the trend was opposite with higher levels of P_2_O_5_ applied to soil. The highest AE_P_ was 30.2 kg kg^-1^, 34.3 kg kg^-1^ and 26.8 kg kg^-1^ with the use of N @ 150 kg ha^-1^, P_2_O_5_ @ 25 kg ha^-1^ and K_2_O @ 75 kg ha^-1^. AE_K_ also increased significantly with higher levels of N and opposite for K levels. The AE_K_ was found non-significant under levels of P_._ The highest AE_K_ was 33.5 kg kg^-1^, 28.7 kg kg^-1^, 37.6 kg kg^-1^ from the application of N @ 150 kg ha^-1^, P_2_O_5_ @ 75 kg ha^-1^ and K_2_O @ 25 kg ha^-1^, respectively.

#### Physiological efficiency

**Fig 9:**
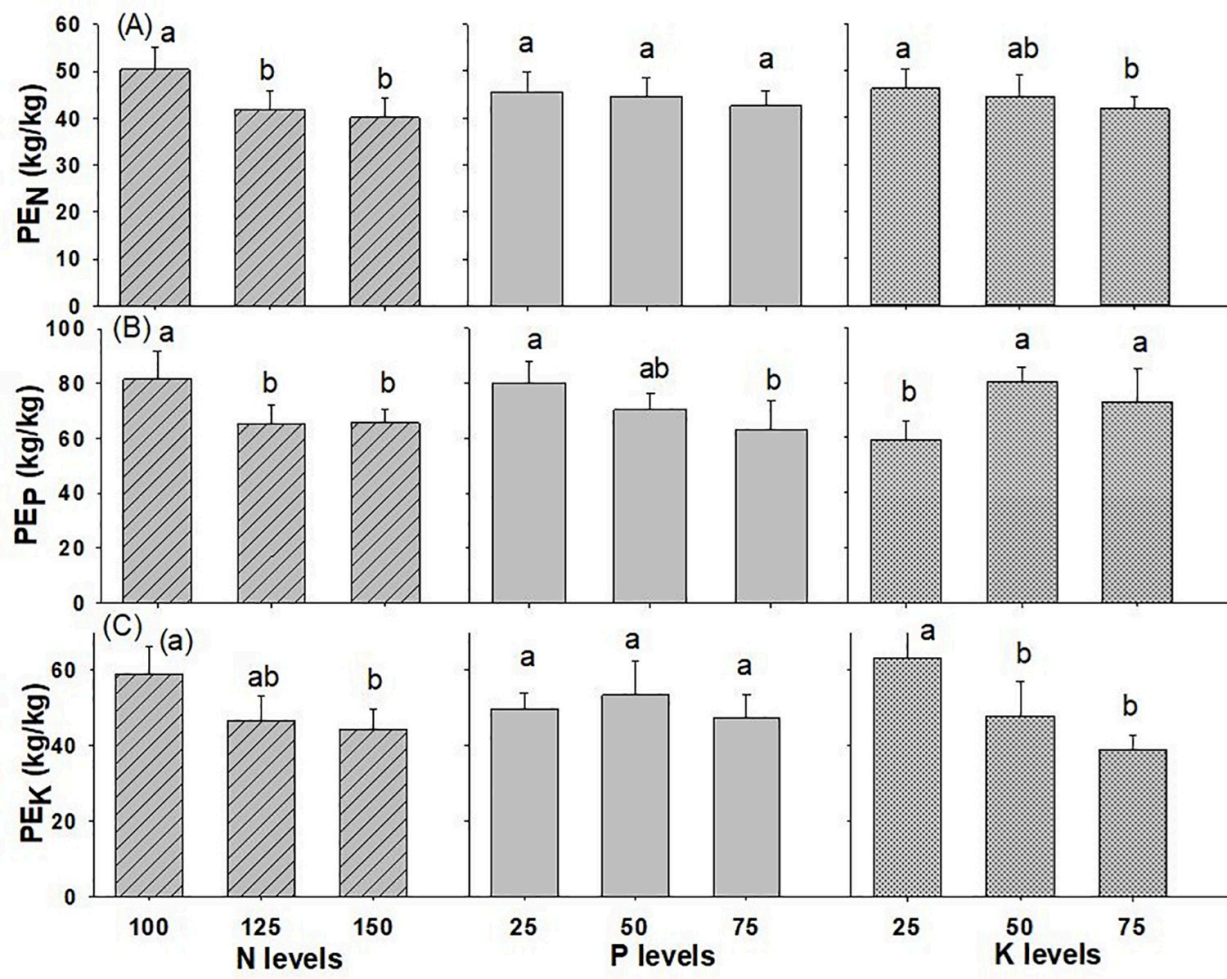
Physiological Efficiency of wheat (A) nitrogen (B) phosphorus (C) potassium as influenced by different levels of NPK fertilizer (2019/20-2020/21). Different small alphabetical letters indicate significant differences at p<0.05 (otherwise statistically at par). Bars indicate mean value of treatments with 95% confidence interval.

The interactions of N x K and N x P x K on PE_P_ were found to be statistically different while all the other interaction effects of N, P, and K on wheat physiological efficiency were found to be comparable, as given in Table 1 and S2 Table. The application of N @ 100 kg ha^-1^, P_2_O_5_ @ 25 kg ha^-1^, and K_2_O @ 25 kg ha^-1^ resulted in a greater PE_N_ of 50.4 kg kg^-1^, 45.4 kg kg^-1^, and 46.2 kg kg^-1^, respectively in which N and K levels showed significant effect whereas the use of P was at par (Fig 9). The use of lower levels of N, P and K gave lower values of PE_N_. In the same way, use of N @ 100 kg ha^-1^, P_2_O_5_ @ 25 kg ha^-1^ and K_2_O @ 50 kg ha^-1^ gave the greater values of PE_P_ (81.5 kg kg^-1^, 80.0 kg kg^-1^ and 80.4 kg kg^-1^, respectively), while the application of N @ 150 kg ha^-1^, P_2_O_5_ @ 75 kg ha^-1^ and K_2_O @ 25 kg ha^-1^ resulted in the lowest PE_P_ (63.6 kg kg^-1^, 62.9 kg kg^-1^ and 59.2 kg kg^-1^, respectively). Likewise, with the use of N @ 100 kg ha^-1^ (59.1 kg kg^-1^), P_2_O_5_ @ 50 kg ha^-1^ (53.3 kg kg^-1^) and K_2_O @ 25 kg ha^-1^ (63.3 kg kg^-1^), the PE_K_ value of wheat was observed the highest, while the lowest value of PE_K_ (44.4 kg kg^-1^, 47.1 kg kg^-1^ and 39.0 kg kg^-1^) were obtained from the application of N @ 150 kg ha^-1^, P_2_O_5_ @ 75 kg ha^-1^ and K_2_O @ 75 kg ha^-1^, respectively.

## Discussion

### Nutrients and grain yield

Since unit cost of fertilizer is expensive as compared to unit price of yield, efficient N fertilizer application is critical for both agroeconomic and environmental reasons [44]. For optimum yield and fertilizer efficiency, fertilizer should be applied at appropriate dose. In both the years, the grain yield of wheat was influenced by N and K levels, with a mean grain yield of 6.27 t ha^-1^. The findings of the study suggested to apply N @ 125 kg ha^-1^ and K_2_O @ 50 kg ha^-1^ as a dose of fertilizer for optimum wheat production which is to be recommended to improve high yielding varieties of wheat with similar day lengths and soil types in mid hill of Nepal. However, there was no considerable rise in phosphorus above 25 kg P_2_O kg ha^-1^ which may be due to higher P availability in the soil. It could be the result of residual P from previous P fertilizer applications that occurred prior to sowing wheat. The average yield in the field experiment was higher as compared to national average yield of wheat which may be with the combined efforts of improved variety, longer crop duration, proper management of irrigation and fertilizers, which provided a significant contribution to the improvement in the yield [45]. The higher experimental wheat yield indicates that the yield seems to have a huge potential to increase, particularly in low-productivity areas. Similar to our findings, when applied simultaneously, N and K have a huge impact on wheat grain production [46]. Likewise, the application of N: P_2_O: K_2_O @ 105:75:75 kg ha^-1^ produced the highest yield [47] while 140 kg N ha^-1^ was recommended for higher grain yield in wheat [48]. Increased N and K application increased nutrient availability in the soil as well as N and K uptake in the wheat plant, resulting in higher wheat grain yields. This could explain why the grain production of wheat was highest when N and K_2_O were applied @ 150 kg ha^-1^ and was 75 kg ha^-1^, respectively. Likewise, a beneficial response of N up to 156 kg ha^-1^ with a grain yield of 6472 kg ha^-1^was observed in irrigated wheat [49]. Similarly, in other studies also, increasing fertilizer levels improved grain yields of wheat significantly [50–53]. Wheat yields were considerably greater when K @ 66 kg ha^-1^ was used compared to 38 kg ha^-1^ [54] while the highest wheat yield was observed with potassium @ 66 kg ha^-1^ [55]. Increased nutrient levels resulted in improved yield attributes, which led to a boost in wheat grain yield.

### Partial factor productivity

The partial factor productivity, which is expressed as crop yield per unit of nutrient provided, is a technique to compare the economic advantages of fertilization. The differences in average PFP between regions are based on yield potential, soil quality, fertilizer application amount and form, and other crop management activities such as overall timeliness and quality [56]. The PFP of N, P and K in wheat were in the range of 43.9–59.0, 85.1–247.3 and 86.4–241.6 kg kg^-1^, respectively is comparable with the values of PFP_N_, PFP_P_ and PFP_K_ (40–90, 100–250, 75–200 kg grain per kg of supplied nutrient, respectively) reported by [57]. The PFP_P_ and PFP_K_ values were considerably greater than the PFP_N_ values. Similarly, the average PFP of N in the world is 44 kg grain per kg N [56], which is consistent with our findings. The mean value of PFP_N_ (51.2 kg kg^-1^) in our research is comparable with the value of 45 kg kg^-1^ in wheat compiled by [19]. PFP_N_ should be between 40 and 70 kg grain per kg N, with more than 70 kg kg^-1^ in in well-managed systems or at low levels of N use [20]. PFP_P_ (152.5 kg kg^-1^) observed in our study was within the line with PFP value of 143 kg kg^-1^ as documented by [40]. Increasing fertilizer rates lowers NUE because yield increases slower than N applied in soil [15].

### Internal efficiency

Internal efficiency is an evaluation of a plant’s ability to convert nutrients from all sources (soil and fertilizer) into grain yield [40]. When IE is very high, it is considered as the deficiency of that particular nutrient and internal nutrient conversion is poor due to other stresses when the IE is low [57]. In this experiment, IE_N_ varied from 46.5 to 52.6 kg kg^-1^, with a mean of 49.0 kg kg^-1^, which is lower than average internal efficiency of the 62.3 kg grain per kg N uptake recorded by [58], within the IE range (18.3–65.9 kg kg^-1^) described in cereal-based systems [31] and similar to the optimal range of IE_N_ (55 to 65 kg kg^-1^) for balanced nutrition at greater yield [22]. Similarly, the values of IE_P_ (100.9 kg kg^-1^) found in our findings were lower than the value of IE_P_ (290.4 kg kg^-1^) recorded [58]. Likewise, the mean IE_K_ value of 33.4 kg kg^-1^ was observed in wheat [58] is lower than the value recorded in our study (42.5 kg kg^-1^).

### Partial nutrient budget

The partial nutrient budget is the description of a removal-to-use ratio. A PNB close to 1 is commonly believed to indicate that soil fertility will be sustained at a constant level. PNB should be greater than one in nutrient-deficient soil, less than one in nutrient-surplus soil, and near to one in sustainable soils [39]. With mean values of 1.1 kg kg^-1^, 1.5 kg kg^-1^ and 3.6 kg kg^-1^, PNB_N_, PNB_P_, and PNB_K_, respectively, were greater when corresponding N, P, and K application rates were low and dropped as nutrient levels increased. The average PNB_N_ in our study was comparable to the value of 1.01 kg kg^-1^ recorded in Montana during 2007 [59]. However, these values were greater than the benchmark values of 0.7–0.9 kg kg^-1^ reported in cereals [57] and the PNB_K_ value of 1.82 kg kg^-1^ obtained [40] which means there was mining of nutrient in the soil. PNB values greater than one indicates the need for fertility replenishment from N, P, and K fertilization [41]. Our study revealed that nutrient uptake was more than the amount of nutrient given through the fertilizer, indicating that the system may not be sustainable. To make it sustainable, extra fertilizer should be used and nutrient loss should be reduced through management measures. The farms with adequate access to resources will have PNB values less than 1 (nutrient input surpasses removal), whereas those with fewer resources will have PNB values more than 1 [60].

### Recovery efficiency

Recovery efficiency is a tool used to investigate the crop’s nutritional response. It is the difference in the uptake of above-ground plant parts between treated and non-treated crops in proportion to the applied nutrient. It is the most useful indicators for analyzing the cumulative effects of N treatment on N availability [61]. In this study, mean RE_N_ was 58.4%. which fall within the range of 40–60% given [57], is similar to RE_N_ of 58% recorded from the use of N @ 120 kg ha^-1^ [31] and is lower than the highest apparent nitrogen recovery efficiency of 68% obtained from the application of N @ 120 kg ha^-1^ [12]. Similarly, wheat recovery efficiency for N fertilizers under favorable weather and unfavorable weather were 49% and 18%, respectively from on farm experiments in India [62]. According to a review of global data on nutrient use efficiency for cereal crops from researcher-managed experimental fields, the average RE_N_ for wheat was 57% [19] which is similar to the results obtained in our research. RE for N should be within 30% to 50%and with low N fertilizer amounts and in well-managed systems or a low soil N supply, RE might reach 50% to 80% [22]. Likewise, NUE of wheat declined when N fertilization levels increased [63, 64]. Similar to the results obtained in this study, [N fertilization enhanced RE_N_ up to certain level, however the greatest N level reduced RE_N_ [65]. RE_N_ in our study first increased to a peak value at 125 kg N ha^-1^ and then decreased with further increased in N dose which showed similar trend as RE_P_ observed [66]. Similarly, increased in RE_N_ for wheat was observed @ 110 N ha^-1^ as compared to 60 and 85 kg N ha^-1^ [67]. This is contrast with the result obtained in which there was declined in RE_N_ with the increase in nitrogen levels from 120 to 360 kg N ha^-1^ [12].

The recovery of applied fertilizer P in the first year varies from less than 10% to as much as 30% [68]. Since, P is immobile in the soil and reactions with other soil minerals are gradual, long-term P recovery by subsequent crops can be considerably higher. The average RE_P_ in our study was 32.7% which is comparable to the RE_P_ of 37.5% obtained [66] and RE_P_ of 27% recorded [31] from the use of phosphorus @ 26 kg ha^-1^. Even though K is stable in most soils and is not prone to the gaseous losses that N is or the fixation reactions that affect P, it is typically thought to have a better use efficiency than N and P. The average RE_K_ in our experiment was 64.8% in wheat which was higher than RE_K_ of 51% obtained from the use of potassium @ 50 kg ha^-1^ [31], and 56.6% apparent recovery efficiency of K recorded [69]. Likewise, the first year’s RE_K_ can be within the range of 20% and 60% [68]. Our results showed that lowest levels of K provided the highest recovery efficiency of K similar to the results obtained by [66]. There was decreasing trend of K recovery with increasing K levels, similar to results observed by [70]. The wheat crop used most of the provided nutrients at lower doses, but at larger doses, the crop failed to use the nutrients effectively which caused lower recovery at higher potassium levels.

### Agronomic efficiency

Agronomic efficiency corresponds to an increase in yield per unit of applied nutrients. The AE_N_ measured in our study ranged from 22.1 to 26.4 kg kg^-1^ with the mean value of 24.3 kg grains kg N^-1^ is lower than the value (36.6 kg kg^-1^) recorded by [66] and similar to the highest agronomic efficiency of 26.4 kg kg^-1^ with N @ 120 kg ha^-1^ observed [12]. Similarly, the average AE_N_ for wheat is suggested to be around 20–25 kg grain increase kg^-1^ N applied globally [65]. Normally, AE for N should be within 10 and 30 kg kg^-1^ and with lower quantities of N fertilizer application and the optimum nutrient management, AE should be larger than 30 kg kg^-1^ [22]. Similarly, the decrease in AE_N_ was recorded with increase in N levels from 30 to 120 kg N ha^-1^ [71]. Likewise, highest AE_N_ was obtained with lower rate of N because of minimized losses [72]. The wheat plant can utilize most of the N supplied for grain production at the lower rate. T AE_N_ was substantially reduced in the highest N fertilizer level, which is similar to the data recorded by various researchers [12, 73]. AE_P_ recorded in the range of 13.2 to 34.3 kg kg^-1^ in wheat was comparable with the RE of P (15–40 kg kg^-1^) suggested [57]. The AE for N, P and K in our study declined as the level of corresponding nutrients increased similar to trend AE obtained [66]. AE for K recorded in the study range from 18.7 to 37.6 kg kg^-1^ with mean value of 27.3 kg kg^-1^ is comparable with the maximum AE_K_ (29.9 kg kg^-1^) observed [69]. AE_K_ decreased with the increase of K rate which is similar to results recorded by [27, 74].

### Physiological efficiency

PE represents an increase in yield per unit of increased crop nutrient uptake in the plant’s above-ground parts. PE_N_ should be between 30 and 60 kg kg^-1^, and it might be greater than 60 kg kg^-1^ in well-managed systems or limited soil N supplies or low levels of N use [20]. In our study, the PE_N_ was poor for the treatments where N, P and K application was high and vice-versa. It ranged from 40.2 to 50.4 kg kg^-1^ which is comparable to the PE_N_ ranged of 35 to 71 kg kg^-1^ obtained by [61], average value of 41 kg kg^-1^ observed in different parts of world [19] and is near about the maximum PE_N_ (46.6 kg kg^-1^) recorded from the application of N @ 120 kg ha^-1^ [12]. The low PE for N suggests that nutrient accumulation (input) was greater than grain production (output). The higher PE_N_ was observed when nutrient supply was low and it decreased as nutrient levels increased [12, 66], which is similar to our findings. PE_P_ range of 59.2 to 81.5 kg kg^-1^ was observed in our study which is lower than the value of PE_P_ (159.7 to 184.1 kg kg^-^1) obtained [66]. Similarly, PE_K_ ranged from 39.0 to 63.3 kg kg^-1^ observed in wheat is comparable to PE_K_ range of 45.0 to 48.9 kg kg^-1^ recorded by [66] and PE_K_ range of 49 to 96 kg kg^-1^ obtained by [61] and lower than the maximum PE_K_ of 83.1 kg kg^-1^ recorded by [69]. Similar to our findings, the highest PE was recorded with the application of lower level of K [74, 75].

## Conclusions

Knowledge of the appropriate fertilizer rate and crop nutrient requirements is critical for farmers to enhance crop yields and nutrient use efficiency. Improving nutrient efficiency is a noble goal as well as a serious issue for the agriculture and fertilizer business. The finding showed that N @ 125 kg ha^-1^, P_2_O_5_ @ 25 kg ha^-1^ and K_2_O @ 50 kg ha^-1^ were the optimum recommendations with higher grain yield and efficient use of nutrients in wheat. The partial factor productivity, internal efficiency, partial nutrient budget, recovery efficiency, agronomic efficiency, and physiological efficiency of NPK for wheat were statistically influenced by nutrient levels. The nutrient use efficiency for N, P, and K decreased with the increase of corresponding dose of nutrients in mid-hills of Nepal. The application of a higher rate of inorganic P and K fertilizer improved wheat N efficiencies, and the case was true for P and K efficiencies. These findings may apply to other locations with similar cropping systems, soil, and climate circumstances for establishing successful nutrient management strategies. The reference value of nutrient use efficiency indices recorded in wheat in this study can be used to quantify the crop response to applied nutrient and minimize nutrient losses for better management practice.

## Supporting information

S1 Table Partial Factor Productivity (PFP), Partial Nutrient Budget (PNB), and Internal Efficiency (IE) of nitrogen, phosphorus and potassium in wheat at Khumaltar, Lalitpur, 2019/20-2020/21 (two years pooled mean). (PDF)

S2 Table Physiological Efficiency (PE), Recovery Efficiency (RE), and Agronomic Efficiency (AE) of nitrogen, phosphorus and potassium in wheat at Khumaltar, Lalitpur, 2019/20-2020/21 (two years pooled mean). (PDF)
