# Supplementary material for: Nutrient use efficiency (NUE) of wheat (Triticum aestivum L.) as affected by NPK fertilization
Source: PLoS One. 2022 Jan 27;17(1):e0262771. doi: 10.1371/journal.pone.0262771 (PMC8794114; doi:10.1371/journal.pone.0262771)
Supplement: S1 Table — (PDF) [file pone.0262771.s001.pdf]

S1 Table: Partial Factor Productivity (PFP), Partial Nutrient Budget (PNB), and Internal Efficiency (IE) of nitrogen, phosphorus and potassium in wheat at Khumaltar, Lalitpur, 2019/20-2020/21 (two years pooled mean)

	Nutrient Use Efficiency (PFP) (kg/ha)			Nutrient Uptake Efficiency (PNB) (kg/ha)			Nutrient Utilization Efficiency (IE) (kg/ha)		
**Treatments**	N	P	K	N	P	K	N	P	K
**Nitrogen levels (N)**									
**100**	59.04	142.8	141.92	1.14	1.30	3.13	52.56	109.52	45.43
**125**	50.64	154.1	153.51	1.07	1.56	3.64	47.99	98.70	41.81
**150**	43.92	160.6	158.58	0.96	1.67	3.96	46.52	94.70	40.16
**LSD 0.05**	1.699	5.03	4.408	0.04	0.072	0.176	1.675	3.815	2.078
**Phosphorus levels (P)**									
**25**	50.37	247.3	149.11	1.02 b	2.38	3.50	49.77	104.84	43.02
**50**	51.09	125.1	151.75	1.05 b	1.26	3.56	49.28	100.57	42.61
**75**	52.13	85.1	153.14	1.10 a	0.88	3.68	48.02	97.51	41.76
**LSD 0.05**	NS	5.03	NS	0.049	0.072	Ns	NS	3.815	NS
**Potassium levels (K)**									
**25**	49.26	147.0	241.59	0.98	1.46	5.59	50.52	101.51	43.89
**50**	51.47	153.1	126.02	1.06	1.51	2.99	49.15	100.99	42.87
**75**	52.87	157.4	86.40	1.13	1.56	2.16	47.40	100.42	40.62
**LSD 0.05**	1.699	5.03	4.408	0.049	0.072	0.176	1.675	NS	2.078
**F test Prob (P>F)**									
**CV (%)**	8.7	8.6	7.6	12.2	12.5	12.9	9.0	9.9	12.8
**Grand Mean**	51.20	152.5	151.34	1.06	1.51	3.58	49.03	100.97	42.46
