# Supplementary material for: Nutrient use efficiency (NUE) of wheat (Triticum aestivum L.) as affected by NPK fertilization
Source: PLoS One. 2022 Jan 27;17(1):e0262771. doi: 10.1371/journal.pone.0262771 (PMC8794114; doi:10.1371/journal.pone.0262771)
Supplement: S2 Table — (PDF) [file pone.0262771.s002.pdf]

S2 Table: Physiological Efficiency (PE), Recovery Efficiency (RE), and Agronomic Efficiency (AE) of nitrogen, phosphorus and potassium in wheat at Khumaltar, Lalitpur, 2019/20-2020/21 (two years pooled mean)

	Physiological Efficiency (PE) (kg/ha)			Recovery Efficiency (RE) (%)			Agronomic Efficiency (AE) (kg/ha)		
**Treatments**	N	P	K	N	P	K	N	P	K
**Nitrogen levels (N)**									
**100**	50.39	81.5	59.1	56.76	14.5	36.8	26.36	13.20	18.74
**125**	41.96	65.4	46.6	60.88	36.5	67.8	24.49	23.62	29.53
**150**	40.18	65.6	44.4	57.53	47.1	89.9	22.13	30.19	33.52
**LSD 0.05**	2.843	14.30	12.93	NS	6.35	11.96	11.699	4.804	4.097
**Phosphorus levels (P)**									
**25**	45.38	80.0	49.6	55.31	44.4	58.1	23.50	34.28	25.42
**50**	44.62	70.2	53.3	57.64	29.8	65.6	24.22	18.45	27.66
**75**	42.52	62.9	47.1	62.21	23.7	70.8	25.26	14.28	28.72
**LSD 0.05**	NS	14.30	NS	4.774	6.35	NS	NS	4.804	NS
**Potassium levels (K)**									
**25**	46.21	59.2 b	63.3	51.27	27.7	76.0	22.39	17.65	37.62
**50**	44.46	80.4 a	47.8	58.68	32.2	60.8	24.60	22.61	24.88
**75**	41.85	72.9 a	39.0	65.22	38.1	57.7	26.00	26.75	19.30
**LSD 0.05**	2.843	14.30	12.93	4.773	6.35	11.96	1.699	4.804	4.097
**F test Prob (P>F)**									
**CV (%)**	8.3	8.7	15.5	21.4	26.9	28.3	18.3	26.4	29.4
**Grand Mean**	66.13	72.56	25.65	58.39	32.7	64.8	24.33	22.34	27.26
